# The proteomic architecture of clonal hematopoiesis: a systematic review of niche remodeling and multi-compartment predictors of malignant transformation

**DOI:** 10.3389/fmed.2026.1818220

**Published:** 2026-08-27

**Authors:** Jana H. Husein, Rasha M. Khayata, Aisha Jahangir, Ghaith K. Mansour, Ahmed W. Hajjar, Muhammad Raihan Sajid

**Affiliations:** 1 College of Medicine, Alfaisal University, Riyadh, Saudi Arabia; 2 Department of Pharmacology, College of Medicine, Alfaisal University, Riyadh, Saudi Arabia; 3 Department of Pathology, College of Medicine, Alfaisal University, Riyadh, Saudi Arabia

**Keywords:** biomarker discovery, bone marrow microenvironment, clonal hematopoiesis, inflammaging, malignant transformation, proteomics

## Abstract

**Background:**

Clonal hematopoiesis of indeterminate potential (CHIP) is a precursor to myeloid malignancies, yet the functional proteomic landscape that governs clonal fitness and microenvironmental remodeling remains poorly understood. This systematic review consolidates high-resolution proteomic evidence to map the transition from stable CHIP to overt malignancy.

**Methods:**

Following PRISMA 2020 guidelines, we analyzed 15 high-quality studies (selected from 83 initial records) published between 2015 and 2025. The review integrates data from diverse proteomic platforms—including DIA-MS, TMT labeling, and Olink high-plex panels across hematopoietic stem/progenitor cells (HSPCs), the bone marrow niche, and blood. Risk of bias was rigorously assessed using ROBINS-E, QUIPS, and SYRCLE frameworks.

**Results:**

Synthesis of the evidence reveals a profound “Genotype-Proteotype Gap,” characterized by a low median mRNA-protein correlation (0.30) in aging stem cells. Clonal expansion is associated with a non-linear “functional niche failure” exceeding a 5% VAF threshold, marked by the emergence of inflammatory mesenchymal stromal cells (iMSCs) expressing IL-1R1 and CD44. Systemically, proteomic risk scores utilizing markers such as F7, BIN2, and CXCL11 can predict incident myeloid neoplasms over a decade before clinical diagnosis. Evidence strength varies substantially, with high-strength support for plasma predictive signatures and preliminary-to-moderate support for mechanistic findings from smaller cohorts.

**Conclusion:**

This systematic review identifies that malignant progression is dictated by a qualitative proteomic “switch” and structural niche reorganization. However, causal relationships remain to be definitively established. We identify a critical “Spatial Architecture Gap” in current research, highlighting the need for spatial proteomic platforms to map the physical “handshake” between mutant clones and their inflammatory environment to enable early clinical interception.

## 1 Introduction

The bone marrow (BM) is an extraordinarily complex and dynamic tissue in which hematopoietic stem and progenitor cells (HSPCs) reside, self-renew, and differentiate. Much like other specialized organ systems, the BM niche is composed of diverse cellular and molecular components whose architecture and microenvironment evolve across the lifespan. These include mesenchymal stromal cells, immune cell subsets, extracellular matrix elements, and soluble signaling factors, all of which work together to maintain hematopoietic homeostasis. However, clonal hematopoiesis of indeterminate potential (CHIP) has emerged as a prevalent age-associated phenomenon characterized by the acquisition of somatic mutations in HSPCs without overt hematologic malignancy or significant cytopenias (1). CHIP is clinically relevant because it increases the risk of developing myeloid neoplasms such as myelodysplastic syndromes (MDS) and acute myeloid leukemia (AML), as well as contributing to age-associated clonal expansion and hematopoietic dysfunction. The progression from CHIP to overt malignancy is thought to occur through gradual remodeling of the BM niche and the acquisition of permissive biological programs within mutant HSPCs (2).

Traditionally, investigations of CHIP biology and progression have relied heavily on genomic sequencing, bulk transcriptomics, and phenotypic assays of isolated HSPCs or stromal cell populations. While these approaches have substantially advanced our understanding of mutational drivers and transcriptional reprogramming, they provide an incomplete picture of the functional consequences of clonal expansion. Proteins rather than genes mediate metabolism, intracellular signaling, immune regulation, and cell to cell communication. Thus, genomic studies alone fail to fully represent post-transcriptional, post-translational, and microenvironmental effects that shape clonal behavior (3, 4).

Recent work has identified altered inflammatory and immune signaling in CHIP-associated bone marrow microenvironments, suggesting that dysfunctional stromal and immune interactions may facilitate HSPC clonal dominance. Inflammatory mesenchymal stromal cells (iMSCs) enriched in interferon-related pathways remodel the human BM niche in CHIP and MDS, altering HSPC behavior and suppressing healthy hematopoiesis (5). Single-cell multiomic studies integrating transcriptomic and protein-level readouts also demonstrate that CHIP-mutant HSPCs exhibit altered responses to inflammatory stress, with attenuated activation signatures that may confer a selective advantage under chronic inflammation and aging (6, 7). Additional proteomic, phosphoproteomic, and integrated multiomics studies in MDS and AML, which represent progressive stages along the clonal hematopoiesis–leukemia continuum, reveal disrupted niche support functions, extracellular matrix alterations, and metabolic reconfiguration (4, 8–11), highlighting potential biomarkers and pathways underlying early malignant transformation.

Despite growing recognition of CHIP as a precursor state to myeloid malignancies, no comprehensive synthesis of proteomic evidence exists that integrates protein-level alterations in HSPCs, BM niche components, and early malignant evolution. Existing studies primarily emphasize genomic changes, leaving a critical gap in our understanding of the functional proteomic mechanisms underlying clonal behavior and malignant progression.

This systematic review therefore seeks to consolidate and critically appraise proteomic alterations associated with CHIP. The primary objective is to identify consistent proteomic changes in HSPCs from individuals with CHIP compared with healthy controls. Additional objectives are to characterize proteomic remodeling within the BM microenvironment and to define proteomic signatures distinguishing stable CHIP from cases progressing to myelodysplastic syndromes (MDS) or acute myeloid leukemia (AML). A comprehensive analysis of disease-specific proteomic features and biomarker candidates implicated in clonal expansion, inflammatory niche remodeling, and early leukemic transformation is essential for developing more accurate risk stratification tools, tailored therapeutic approaches, and focused diagnostic strategies for hematologic malignancies.

## 2 Methods

### 2.1 Protocol and registration

This systematic review was conducted in strict accordance with the Preferred Reporting Items for Systematic Reviews and Meta-Analyses (PRISMA 2020) guidelines to ensure transparent and comprehensive reporting (12). The study framework was guided by the PICOS (Population, Intervention, Comparison, Outcome, and Study Design) model as outlined in Table 1. Although the protocol was not formally registered in a public database, all methodological procedures including search strategies, inclusion/exclusion criteria, and data extraction protocols were established prior to starting in a standardized internal protocol. This framework was strictly followed throughout the review process to ensure methodological precision and minimize the risk of post-hoc bias in the selection and synthesis of the proteomic signatures associated with clonal hematopoiesis and its progression to myeloid malignancies. The absence of prospective registration is clearly noted as a limitation in Section 4.6.

**Table 1:** PICOS framework.

PICOS	Description
P (population)	Human subjects with CHIP (with or without progression to hematologic malignancy); relevant animal models of CHIP.
I (intervention)	Proteomic analysis of HSPCs, bulk bone marrow, or bone marrow niche components.
C (comparison)	Healthy control subjects or individuals with CHIP who did not progress to malignancy.
O (outcome)	Differential protein expression, pathway activation (e.g., inflammation, DNA damage response, metabolism), protein-based signatures of progression risk.
S (study design)	Prospective and retrospective longitudinal cohorts, case–control studies, and translational mechanistic animal experiments.

### 2.2 Information sources and search strategy

A comprehensive search strategy was conducted across PubMed, Scopus, and Google Scholar to identify any relevant studies that explore the role of proteomics in unraveling the pathogenesis of Clonal Hematopoiesis of Indeterminate Potential (CHIP) and its progression to hematologic malignancy. PubMed and Scopus were selected as primary indexed databases due to their extensive coverage of biomedical and proteomic literature. Google Scholar was included to enhance capture of gray literature and studies not yet indexed in traditional databases. Embase and Web of Science were not included due to institutional access issues; however, we supplemented our search with manual curation of reference lists from included studies and relevant review articles to mitigate potential omissions. Keywords and Medical Subject Headings (MeSH) terms were combined using Boolean operators (AND/OR) to enhance the search precision. The query included terms such as: (“proteomics” OR “mass spectrometry” OR “Proteome”) AND (“clonal hematopoiesis” OR “CHIP” OR “ARCH” OR “age-related clonal hematopoiesis”) AND (Myelodysplastic Syndromes OR Acute Myeloid Leukemia OR AML OR MDS) (“progression” OR “pathogenesis” OR “microenvironment” OR “bone marrow niche”). Manual curation of reference lists from included studies and relevant review articles was also performed to identify additional eligible literature.

### 2.3 Inclusion and exclusion criteria

The inclusion criteria comprised of original, peer-reviewed research articles published in English between 2015 and 2025. This timeframe was established to prioritize high-sensitivity proteomic platforms and to ensure that the human cohorts adhered to modern diagnostic consensus for clonal hematopoiesis. Specifically, the review included: (1) studies performing untargeted or targeted proteomics on samples from defined CHIP cohorts; (2) studies investigating the proteomic interface between CHIP and established malignancies (MDS, AML, MPN), where CHIP represented a precursor state; and (3) studies of overt myeloid malignancies (AML, MDS, PV, myelofibrosis) that provided mechanistic insights into the transition from pre-malignant to malignant states through proteomic characterization of disease progression. For studies in category (3), we explicitly distinguished findings derived from CHIP-proximal models (e.g., early-stage disease, mutant HSPCs) from those that may reflect consequences of established malignancy rather than mechanisms of transformation. This distinction is highlighted throughout the Results and Discussion.

The exclusion criteria included non-peer-reviewed articles, review articles, case reports, editorials, letters to the editor, and studies focusing exclusively on genomic sequencing without accompanying proteomic or functional signaling data. Studies written in languages other than English or published before 2015 were also excluded.

### 2.4 Study selection

The selection process was conducted in multiple phases to ensure a rigorous and unbiased identification of relevant literature. Following the execution of the search strategy, all identified records were consolidated and exported to MyBib for centralized management and systematic de-duplication. This automated reconciliation identified and removed redundant records across PubMed, Scopus, and Google Scholar prior to the screening phase (13).

The screening was performed independently by three reviewers in two distinct stages. In the first stage, titles and abstracts were assessed for relevance against the predefined PICOS framework. Records that did not meet the primary inclusion criteria were excluded at this level. In the second stage, the full-text versions of potentially eligible studies were retrieved and reviewed in detail to confirm suitability for data extraction.

To maintain a high level of rigor, any discrepancies between reviewers regarding study eligibility were resolved through consensus. The final list of included studies and the specific reasons for the exclusion of full-text articles were documented for inclusion in the PRISMA flow diagram.

### 2.5 Data extraction

Data extraction was performed independently by three reviewers using a standardized data extraction form developed in Microsoft Excel. Variables of interest included: study characteristics (author, year, design, sample size); population demographics (age, disease subtype); biological compartment (e.g., plasma, HSPCs, bone marrow stroma); methodology (proteomic platform, protein coverage, sequencing depth/panel size); key proteogenomic findings (mutational landscape/VAF); and significant proteomic biomarkers (up-and downregulated proteins and functional pathways). The extraction template was pilot tested on a subset of included studies to ensure consistency between reviewers. Any discrepancies regarding extracted data were resolved through discussion.

### 2.6 Risk of bias assessment

To ensure methodological rigor and reliability of the synthesized evidence, a comprehensive risk of bias (RoB) assessment was performed independently by two reviewers for the included studies. Given the methodological heterogeneity of the eligible literature, which comprised both human epidemiological cohorts and translational murine models, three validated risk-of-bias tools were employed. Each tool was selected according to the specific study design it was intended to evaluate. Studies incorporating both human observational data and murine models were assessed using two appropriate tools to ensure complete and design-specific evaluation. To facilitate transparent reporting, the resulting RoB assessments were synthesized and visualized using the robvis website (14).

The methodological quality of the twelve studies providing human observational and cross-sectional evidence was assessed using the Risk of Bias in Non-randomized Studies of Exposures (ROBINS-E) framework (15). This validated tool enables a structured evaluation across seven predefined domains: bias due to confounding, selection of participants into the study, classification of exposures, departures from intended exposures, missing data, measurement of outcomes, and selection of the reported result. Application of the ROBINS-E framework allowed for a systematic appraisal of internal validity and facilitated transparent identification of potential sources of bias that could influence the reported proteomic signatures associated with clonal hematopoiesis and myeloid disease states.

For studies investigating the longitudinal relationship between proteomic biomarkers and clinical progression in human cohorts, the Quality In Prognostic Studies (QUIPS) tool was used (16). This validated framework is the gold standard for evaluating risk of bias in prognostic factor research and comprises six critical domains: 1-study participation, 2-study attrition, 3-prognostic factor measurement, 4-outcome measurement, 5-adjustment for study confounding, and 6 statistical analysis and reporting.

The reliability of mechanistic evidence derived from murine models was assessed using the SYRCLE Risk of Bias tool, a validated framework specifically adapted from the Cochrane Risk of Bias Tool for the laboratory animal context (17). The assessment covered ten domains across five types of bias: selection, performance, detection, attrition, and reporting bias.

### 2.7 Data synthesis

A quantitative meta-analysis was not feasible due to the substantial methodological heterogeneity among the included studies. The dataset comprised a diverse array of study designs, ranging from human epidemiological cohorts to translational murine models. Furthermore, the reported endpoints varied widely, encompassing epidemiological prevalence (e.g., CHIP frequency), molecular biomarkers (e.g., plasma protein levels, cytokine signatures), and qualitative histological changes (e.g., bone marrow fibrosis, adipogenic differentiation). This variability was further compounded by differences in analytical platforms, such as the use of disparate Next-Generation Sequencing (NGS) panels and proteomic quantification methods. Consequently, pooling statistical estimates would have yielded results lacking biological robustness. Instead, a structured narrative synthesis was conducted using evidence mapping across four biological domains, with explicit grading of evidence strength (Section 3.8), to comprehensively map the mechanistic interplay between clonal hematopoiesis, inflammatory bone marrow niches, and disease progression.

## 3 Results

### 3.1 Study selection and quality

#### 3.1.1 Study selection

**Figure 1:**
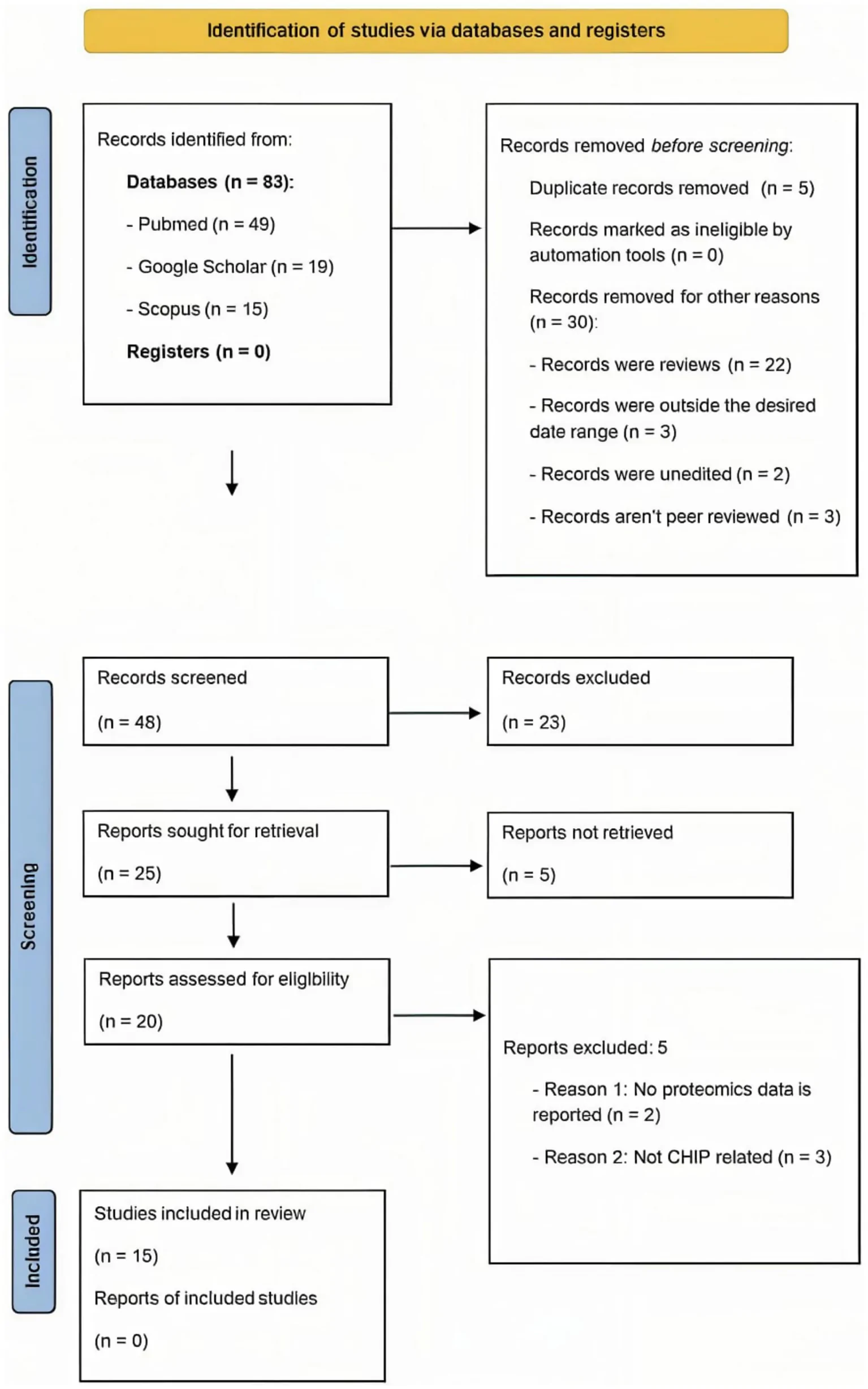
PRISMA flow diagram.

As illustrated in the PRISMA 2020 flow diagram (Figure 1), the systematic search yielded a total of 83 records: PubMed (*n* = 49), Google Scholar (*n* = 19), Scopus (*n* = 15). In the pre-screening phase, 35 records were excluded for the following reasons: duplicate entries (*n* = 5), review articles (*n* = 22), publication date was outside the 2015–2025 range (*n* = 3), non-peer reviewed status (*n* = 3), and publications were preprinted (*n* = 2).

The remaining 48 records underwent initial title and abstract screening, resulting in the exclusion of 23 articles due to lack of relevance to the study objectives. The full texts of the remaining 25 articles were sought for retrieval, of which 5 records were inaccessible. The remaining 20 articles were meticulously evaluated against the predefined eligibility criteria. This secondary screening led to the exclusion of 5 additional studies: two articles did not report proteomics data, and three articles were not CHIP related. Ultimately, 15 studies were retained for qualitative synthesis and risk of bias assessment.

#### 3.1.2 Study characteristics

This systematic review encompasses 15 studies published between 2015 and 2025 that investigate the proteomic interface between CHIP and hematological malignancies (AML/MDS). The research designs include large-scale human cohort studies, cross-sectional analyses, and mechanistic murine models. To capture the functional proteome, the included studies utilized a range of high-sensitivity analytical platforms, including mass spectrometry with quantitative workflows such as Data-Independent Acquisition (DIA-MS), Tandem Mass Tag (TMT) labeling, and Label-Free Quantification (LFQ). High-plex proteomics approaches were also employed, using both targeted and discovery-based scaling through the Olink Explore 1,536 platform and SomaScan secretome profiling. In addition, single-cell proteogenomics techniques were applied, integrating protein and genomic readouts via TARGET-seq+, the Mission Bio Tapestri platform, and the GoTChA assay. The research primarily focused on identifying protein biomarkers and signaling shifts across three biological compartments: hematopoietic stem and progenitor cells (HSPCs), the bone marrow microenvironment (including mesenchymal stromal cells and immune subsets), and plasma.

#### 3.1.3 Population and demographics

The combined population, as seen in Table 2, across these studies spans a wide range of ages and clinical conditions. The total human participants exceeded 427,722, largely driven by massive biobank datasets, complemented by small-scale, deep-phenotyping clinical cohorts.

**Table 2:** Population categories and their definitions.

Population category	Definitions
Healthy controls	Neonatal cord blood, young volunteers (ages 18–35), and age-matched donors screened for the absence of somatic driver mutations (VAF ≤ 3%) and inflammatory markers like C-reactive protein.
CHIP/pre-malignancy	Individuals with somatic mutations (e.g., *DNMT3A, TET2*) with a Variant Allele Fraction (VAF) typically ≥ 2%, consistent with established clinical definitions of CHIP, though reaching ≥ 9% in high-burden cases.
MDS patients	Includes treatment-naïve patients stratified into low-risk (MDS-RS) and high-risk (MDS-EB) subtypes.
AML patients	Primarily de novo AML cases, including various cytogenetic and mutational subtypes (e.g., NPM1, FLT3-ITD).

These human findings were complemented by mechanistic validation in murine models (e.g., C57BL/6 and transgenic JAK2V617F models) to isolate cell-autonomous mechanisms difficult to resolve in human clinical cohorts. Summary of included studies can be visualized in Table 3.

**Table 3:** Summary of included studies.

Study and author	Population			Key proteomic signatures and findings	Functional and clinical impact
	No. of participant	Participating cohort	Age		
Weickert et al. (8)	*N* = 73	Healthy (21), MDS (22), AML (30)	Healthy 20–78 MDS 40–87 AML 21–84	downarrow DLK1 expression Reduced frequency (7 to 45-fold) of CD271 + BMSC subpopulations	Niche Failure: Shift toward a “fatty marrow” phenotype with increased adipogenic potential
Passaro et al. (9)	Mouse Model + Human Cells	8 AML patients + 8 Healthy donors	AML 24–72 Healthy full-term new borns	uparrow RAF/MEK/ERK, PI3K/AKT signaling Downregulation of innate immunity and clotting proteins	Niche Remodeling: Identification of early biomarkers and the compensatory role of Neshigh mesenchymal cells
Tran et al. (24)	*N* = 427,722	UK Biobank (primary and validation)	Mean age:61	Causal Markers: F7 and IL17RA Top risk proteins: BIN2, FGF2, LY9, CXCL11, SORT1	Risk Stratification: Proteomic risk score (AUC 0.85) significantly outperforms standard clinical models
Raffel et al. (4)	*N* = 26	AML = 14 Proteome Healthy HSPCs = 9 RNA Seq Healthy HSPCs = 3 Mice 130	AML:23–74 Healthy: 60–75	LSC Markers: MBOAT7, CRIP2, GNG11, SESN1	Metabolic Vulnerability: LSCs rely on OXPHOS and specific lipid metabolism not detected at the mRNA level
Kramer et al. (18)	*N* = 50	44 AML (*de novo*), 6 Healthy controls	Adults age not specified	uparrow KDMA4/B/C (*IDH1/2* mutant) uparrow phosphorylation of FGR and HCK (FLT3-TKD)	Therapeutic Targets: Identified CD180 and MRC1/CD206 as candidates with minimal healthy expression
Moura et al. (19)	*N* = 66	Discovery (28) and validation (45) cohorts	42–92	uparrow TLN1, MSN, UBC (MDS-EB plasma) uparrow CEP55 (MDS-RS and abnormal karyotypes)	Disease Progression: TLN1 and CEP55 established as signatures for MDS stratification and prognosis
Ismail et al. (7)	*N* = 243	COVID-19 cohort (30% *DNMT3A*mt, 28% *TET2*mt)	Mean 58.5 (±17.5)	uparrow IL-32 (Lymphoid cells) \uparrow Serum MCP-1 (*DNMT3A*mt)	Clinical Morbidity: *DNMT3A*mt as an independent factor for ARDS and overall mortality
Jakobsen et al. (6)	*N* = 195	CH with High VAF (57), low VAF (28), controls (110)	30.9–92	uparrow CD38/CD45RA, downarrow CD49f (*TET2*mt) uparrow A20 (TNFAIP3) protein	Clonal Advantage: Mutant HSCs exhibit an attenuated inflammatory response, facilitating survival over WT cells
Leimkühler et al. (20)	*N* = 225 Human, 151 Murine	139 MPN patients and 86Healthy controls	Mean MPN patients 60.41 ± 1.05 Mean Healthy controls 53.68 ± 3.13	S100A8/S100A9 Alarmin Complex downarrow CXCL12 and KITL (Stem Cell Factor)	Fibrosis Driver: Alarmin axis drives stromal differentiation into myofibroblasts; druggable by Tasquinimod
Nageswara Rao (21)	N/A	Human Murine	N/A	uparrow PFKFB3 protein downarrow Mitochondrial DNA copy number/mass	Metabolic Syndrome: JAK2 mutations cause systemic hypoglycemia and adipose atrophy; reversible by glycolytic inhibitors
Zaro et al. (26)	Murine study	Immunocompetent C57BL/6 mice	N/A	35% increase in protein diversity (Old HSCs) uparrow vWF and Slamf1 (CD150) in aged HSCs	Post-transcriptional Decoupling: *DNMT3A* protein is absent in quiescent HSCs despite stable mRNA levels
Prummel et al. (5)	*N* = 84	35 Healthy, 17 CHIP, 32 MDS	60–89	iMSC Markers: IL-1R1 and CD44 Loss of Pre-B/Pro-B cell progenitors	Niche Remodeling: Identification of inflammatory MSCs (iMSCs) as drivers of transition from CHIP to MDS
Amon et al. (22)	*N* = 11 healthy donors	Healthy donors	for the first 5= 28–57 For the added 6 the ages are no specified	Stemness: GAR1, DKC1, IDH1/3A/3B uparrow Telomerase maintenance complex proteins	Quiescence Baseline: Established that “stemness-maintaining” clusters are upregulated at protein level despite mRNA downregulation
Boer et al. (23)	No = 165	De novo AML = 110 AML from MDS = 27 MDS = 13 APL = 4 Healthy controls = 11	N/A	uparrow IL1RAP (hallmark of L-GMP state) uparrow Secretion of IL-8 and CXCL1	Inflammatory Niche: The IL1-IL1RAP axis suppresses healthy hematopoiesis to favor leukemic expansion
Tan et al. (25)	*N* = 39	18 PV patients, 21 Controls	PV patients -Chronic = 43–79 -Progressed = 61–82 Control groups -Healthy donors = 45–74 -Hemochromatosis = 40–71	Progression Markers: LGALS9 and SOCS2 downarrow CLU protein (HSC/MPPs)	Kinetic Switch: LGALS9-SOCS2 axis persists despite hydroxyurea treatment, marking active leukemic evolution

### 3.2 Risk of bias

In accordance with the dual-appraisal strategy outlined in the Methodology (Section 2.6), the 15 included studies underwent a total of 20 risk-of-bias assessments to account for both human clinical and murine models.

**Figure 2:**
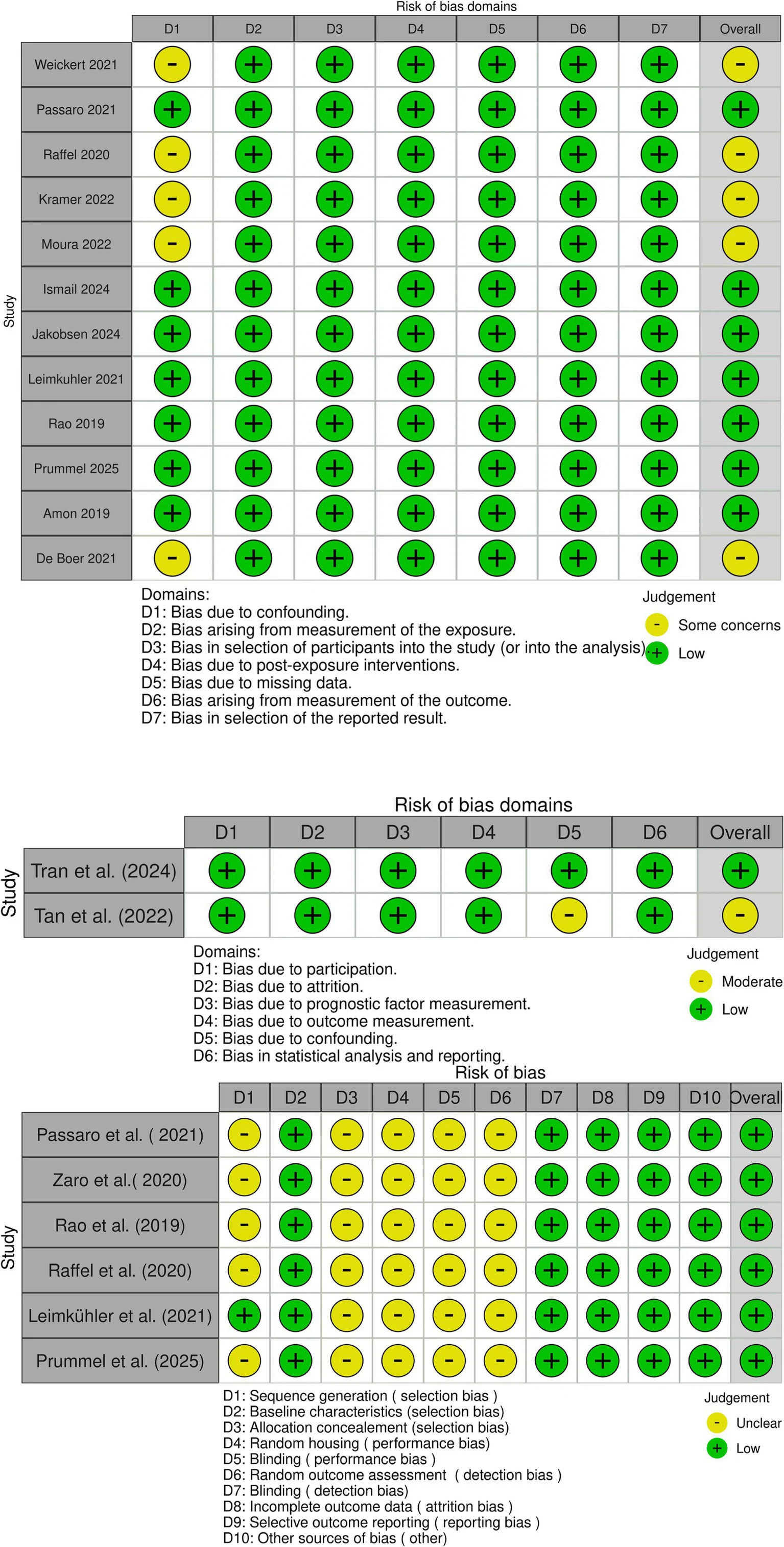
(Panel-1) Risk of bias assessment of included studies. The quality of the studies was evaluated across seven distinct domains using the standard assessment tool ROBINS E. Individual study judgments are indicated by green circles (+) representing a low risk of bias and yellow circles (−) representing some concerns. While most studies demonstrated a low risk across all domains, several were identified as having “some concerns” primarily due to potential confounding factors in domain D1. (Panel-2) Quality in prognosis studies (QUIPS) assessment for prognostic factors. Green circles (+) indicate low risk of bias, yellow circles indicate moderate risk (QUIPS). (Panel-3) SYRCLE’s risk of bias tool for animal studies; domains (D1–D10) cover selection, performance, detection, attrition, and reporting biases. Green circles (+) indicate a low risk of bias, while yellow circles (−) indicate unclear risk.

The risk of bias assessment for 12 of the included studies, visualized in Figure 2 panel 1, was conducted using the ROBINS-E framework. Seven of the studies were evaluated to be low risk of bias, while five were rated as having moderate risk, with the greatest variability observed in domains related to confounding (4–9, 15, 18–23).

All twelve studies were given an overall low risk of bias for domains 2 through 7 because the technical precision was supported by adherence to standardized WHO diagnostic criteria and strict Variant Allele Fraction (VAF) thresholds, alongside the use of high-sensitivity analytical platforms such as: TMT-labeling, DIA-MS, and Olink panels. This ensured that almost no data was missing and eliminated observer bias through automated discovery tools. However, five of the 12 studies received a “moderate risk” for domain 1 (bias due to confounding) (4, 8, 18, 19, 23). These five studies had a restricted sample size making it difficult to account for baseline differences (like age), creating a moderate risk of bias.

For Weickert et al., the concern stems from a relatively small sample size across disease subgroups (Healthy: 21, MDS: 22, AML: 30), which may not sufficiently account for individual biological variance in bone marrow stromal cell subpopulations (8). Similarly, Raffel et al. utilized a limited human cohort of 14 AML patients and 9 healthy controls, which may lead to deep-scale proteomic profiles being influenced by unmeasured participant-specific factors (4). In the case of Moura et al., the global discovery-phase proteomics relied on 28 patients, making it difficult to ensure that the identified signatures were not skewed by the biological diversity of the individual donors (19). Boer et al. was given a moderate rating due to clinical heterogeneity, as the inclusion of both *de novo* and secondary AML (arising from MDS) introduces complex etiological variables that were not fully adjusted for (23). Finally, Kramer et al. compared a wide range of cytogenetic and mutational AML landscapes against an extremely small healthy control group of only six volunteers, which may not provide a representative baseline for comparison (18).

Domain 1 (Bias due to Confounding) was rated low in the remaining seven studies due to the implementation of robust internal controls, such as intra-individual mutant-versus-wild-type comparisons, age-matched cohorts, and dual-species validation models (5–7, 9, 20–22).

Prognostic and longitudinal studies assessed using the Quality in Prognosis Studies (QUIPS) tool was used to evaluate two studies (16, 24, 25). Figure 2 panel 2 demonstrates generally low risk of bias in domains related to prognostic factor measurement and outcome assessment, while variability was primarily observed in the extent of confounding control due to differences in cohort size and study design. Within this framework, Tran et al. was categorized as a prognostic study based on its large-scale longitudinal design using the UK Biobank, in which participants were followed for up to 12 years to predict incident myeloid neoplasm (MN) risk (24). In contrast, Tan et al. was evaluated using QUIPS due to its specific aim of identifying proteomic signatures associated with the clinical transition from chronic polycythemia vera (PV) to progressed disease states (25). Tran et al. Achieved an overall low risk of bias, on the other hand Tan et al. was assigned an overall moderate risk of bias (24, 25).

Domain 5 was critically assessed for the role of clinical variables and sample size. Tran et al. was judged as Low Risk due to its massive cohort *N* = 46,237 and multivariable Cox regression models adjusted for age, sex, smoking, and genetic ancestry (24). Conversely, Tan et al. was assigned a Moderate Risk for confounding; while the study provided high-quality DIA-MS data and functional validation, the relatively small clinical cohort *n* = 18 PV patients restricted the statistical power necessary for exhaustive adjustment of all potential demographic and clinical variables (25).

All remaining domains (Participation, Attrition, Outcome Measurement, and Statistical Reporting) were judged as Low Risk due to the use of well-defined clinical cohorts and objective, externally validated data sources. Specifically, both studies accounted for their entire patient populations throughout the workflow and utilized standardized, peer-reviewed bioinformatic pipelines that ensured transparent and complete outcome reporting. Both studies achieved Low Risk in domain 3 for prognostic factor measurement by utilizing high-precision, automated platforms—Olink Explore 1,536 for Tran et al. and high-sensitivity data-independent acquisition mass spectrometry (DIA-MS) for Tan et al. (24, 25). The use of standardized proteomic workflows and objective machine readouts minimized human interpretive error.

As illustrated in Figure 2 panel 3, the six studies incorporating murine models were evaluated using the SYRCLE Risk of Bias tool (4, 5, 9, 17, 20, 21, 26). Leimkühler et al. was the only study to receive a “Low Risk” rating for selection bias (Domain 1), as it was the only one to explicitly report a randomization sequence (20). In contrast, the other five studies were rated “Unclear” for both sequence generation (D1) and allocation concealment (D3). However, all six studies achieved a “Low Risk” rating for baseline characteristics (D2) by using controls matched for age, sex, and strain. Other areas remained largely vague due to poor documentation: 1 Performance Bias (D4, D5): Most studies were rated “Unclear” because they failed to report on cage randomization, caretaker blinding, or random outcome assessments. 2-Logistical Protocols (D6): This domain was also rated “Unclear” across the board due to insufficient reporting on specific study procedures.

Crucially, all studies achieved a Low-Risk rating for detection bias (D7), as they utilized objective, automated platforms—including high-resolution mass spectrometry, Seahorse XF analyzers, and the Opera Phenix imaging system—effectively eliminating human subjective interpretation. Similarly, attrition (D8), reporting (D9), and other biases (D10) were consistently rated as Low Risk, with stable animal counts and transparent outcome reporting across all figures.

Despite “Unclear” ratings in logistical domains, an overall judgment of Low Risk was assigned to all included studies. This designation reflects the high technical rigor and the use of machine-generated molecular data in critical domains, which lessened the impact of missing data, so the studies stayed accurate.

### 3.3 Proteomic landscape of aging and clonal initiation (stable/low risk CHIP)

**Figure 3:**
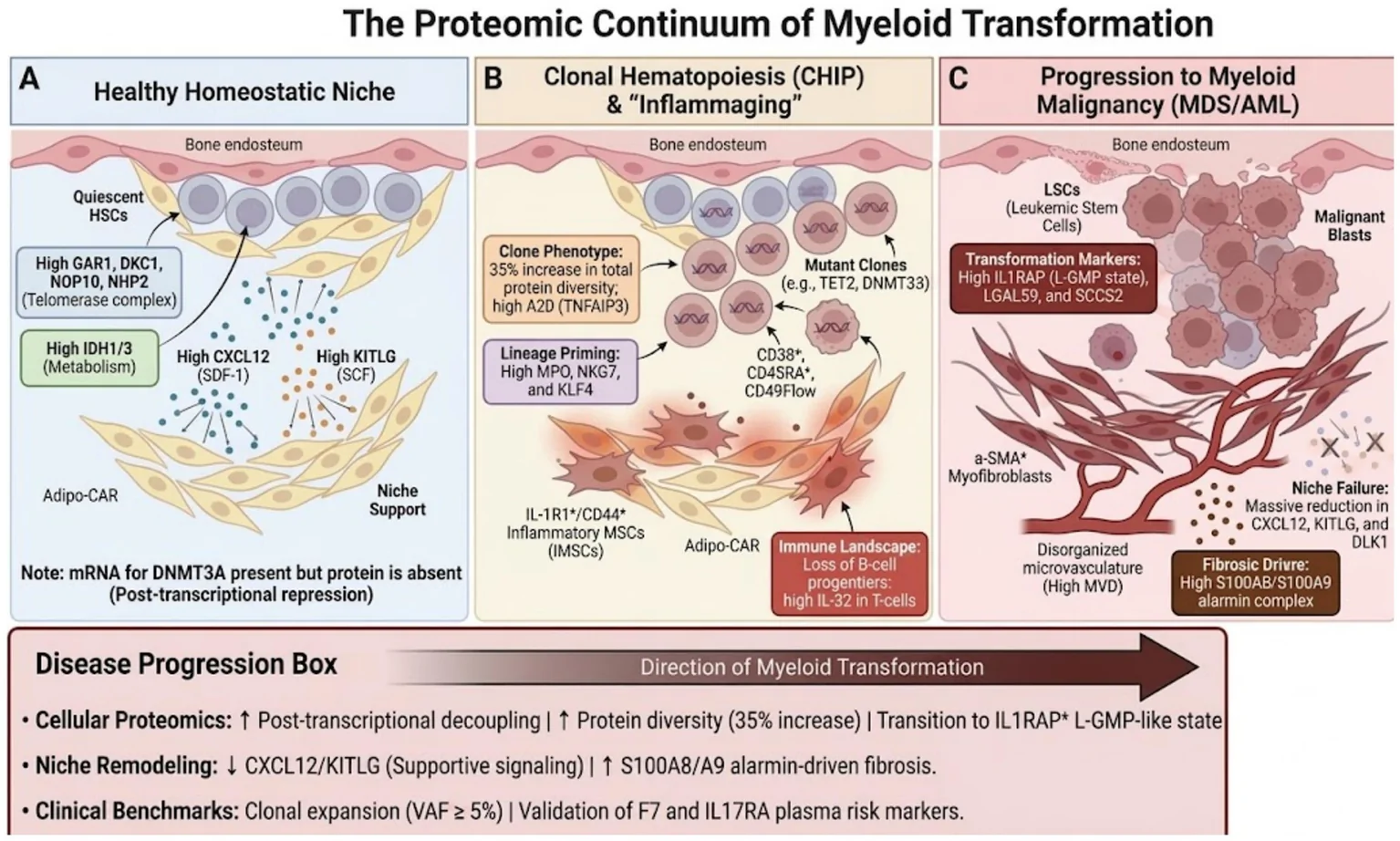
The proteomic continuum of myeloid transformation Figure 3: The proteomic architecture of clonal evolution (image generated by Gemini-Pro version-3). **(A)** The proteomic-transcriptomic gap in aging HSCs: Schematic illustrating the 35% increase in protein diversity in aged HSCs (e.g., GAR1, IDH1) despite stable mRNA levels, highlighting the translational control of *DNMT3A* during cell-cycle entry. **(B)** Niche polarization and iMSC emergence: Depicts the transition of supportive MSCs to inflammatory iMSCs (IL-1R1^+^/CD44^+^) and the subsequent withdrawal of critical niche factors like CXCL12 and KITLG. **(C)** Malignant transformation and the L-GMP phenotype: Illustrates the acquisition of IL1RAP and the IL-1 signaling axis in L-GMP-like cells, driving the suppression of healthy hematopoiesis and niche collapse.

Proteomic analyses demonstrate a marked divergence between transcriptomic and proteomic regulation within the aging hematopoietic system. Aging mouse hematopoietic stem cells (HSCs) exhibit a 35% increase in protein diversity, expressing approximately 5,434 unique proteins compared with 4,030 in younger controls, despite relatively stable mRNA levels (26). This discrepancy is characterized by a low median Spearman correlation (≈ 0.30) between mRNA and protein expression within the HSC compartment. Primarily, *DNMT3A* mRNA is present in quiescent young HSCs; however, the corresponding protein is proteomically undetected and accumulates only upon cell-cycle entry, as illustrated in Figure 3A, (26).

High-sensitivity data-independent acquisition mass spectrometry (DIA-MS) further identified stemness-associated protein clusters, including GAR1, DKC1, and IDH1, which are upregulated at the protein level despite reduced transcript abundance (22). These data indicate substantial post-transcriptional regulation within quiescent HSCs. Within clonal hematopoiesis, mutation-associated proteomic programs demonstrate distinct lineage-associated priming. In stable CHIP with *TET2* mutations, the expression of mature myeloid proteins, including MPO, NKG7, and KLF4, alongside increased CD38 and CD45RA expression and reduced CD49f levels (6). In contrast, *DNMT3A* mutations induce epigenetic transcriptional priming affecting both myeloid and lymphoid lineages, including increased chromatin accessibility at the IL-32 locus and subsequent overexpression of IL-32 in T lymphocytes and NK cells (7). Collectively, these studies indicate that proteomic dysregulation in CHIP reflects post-transcriptional control mechanisms that may precede overt transcriptional reprogramming. This evidence derives primarily from murine models and small human cohorts (Amon et al., *N* = 11) (22, 26); as such, these findings are graded as Moderate-High strength for the proteogenomic discordance phenomenon and Moderate for mutation-specific lineage priming patterns.

### 3.4 Niche remodeling and immune landscape polarization (high-risk CHIP, VAF ≥ 5%)

Niche remodeling occurs following the expansion of clonal burden, particularly at variant allele frequencies (VAF) ≥ 5%, most commonly associated with *DNMT3A* mutations (5). This process is characterized by the emergence of inflammatory mesenchymal stromal cells (iMSCs) defined by increased IL-1R1 and CD44 expression (Figure 3B). The shift from a regenerative to a dysfunctional stromal program is accompanied by reduced expression of key support proteins, including CXCL12, macrophage colony-stimulating factor (M-CSF), and granulocyte–macrophage colony-stimulating factor (GM-CSF) (5, 7).

Immune landscape remodeling is also observed, characterized by the loss of B-cell progenitors and mature B cells alongside an expansion of interferon-responsive CD8^+^ T cells and cytotoxic TEMRA cells (5). Systemically*, DNMT3A*-mutant CHIP is associated with elevated serum MCP-1 levels and increased IL-32 expression in lymphoid cells (7). While these alterations define the CHIP-associated microenvironment, more profound structural changes are reserved for the transition to overt disease; for instance, Weickert et al. demonstrates that the 7 to 45-fold reduction in CD271^+^ bone marrow stromal cells and loss of colony-forming unit-fibroblast (CFU-F) capacity are primary hallmarks of the progression to Myelodysplastic Syndromes (MDS) and Acute Myeloid Leukemia (AML) (8). These niche remodeling findings are supported by moderate-strength evidence from Prummel et al. (*N* = 84, ROBINS-E Low) and Ismail et al. (*N* = 243, ROBINS-E Low), with consistent findings across studies (5, 7).

### 3.5 Proteomic drivers of malignant transformation and progression (progression to MDS/AML)

Progression from CHIP to myeloid malignancy is associated with defined proteomic alterations within hematopoietic stem and progenitor compartments. Increased expression of interleukin-1 receptor accessory protein (IL1RAP) marks a leukemic granulocyte–monocyte progenitor (L-GMP)–like state, representing a critical boundary between clonal expansion and leukemic transformation (23). Activation of the IL-1–IL1RAP axis induces the secretion of inflammatory mediators, including IL-8 and CXCL1, which reduces the proliferation and colony-forming capacity of healthy CD34^+^ cells while favoring leukemic expansion.

Further progression is associated with the activation of an LGALS9–SOCS2 axis within HSC/Multipotent Progenitor (MPP) populations, marking the definitive transition toward leukemic evolution (25). These markers persist despite cytoreductive therapy with hydroxyurea. This malignant transformation is accompanied by the complete collapse of marrow architecture, characterized by the previously mentioned 7 to 45-fold reduction in CD271^+^ stromal cells and loss of CFU-F capacity (Figure 3C) (8). NES-high mesenchymal cells have been reported to provide partial compensatory support during this structural decline (9). These convergent inflammatory and stromal alterations suggest that malignant progression is driven by coordinated intrinsic and extrinsic proteomic remodeling. Evidence for these transformation drivers is graded as Preliminary for IL1RAP [(23), ROBINS-E Moderate] and Preliminary-Moderate for LGALS9-SOCS2 [(25), QUIPS Moderate], reflecting the small sample sizes of the supporting cohorts and the need for replication.

### 3.6 Predictive plasma proteomic signatures and clinical risk stratification (pre-diagnostic/high-risk CHIP)

Systemic proteomic alterations preceded the clinical diagnosis of myeloid neoplasms by more than a decade. A plasma proteomic risk score demonstrated improved predictive performance compared with clinical and genetic models alone (AUC 0.85 vs. 0.80). Mendelian randomization identified coagulation factor VII (F7) and IL17RA as causal protein markers associated with disease progression (24). Additional proteins associated with future myeloid neoplasm risk included BIN2, FGF2, LY9, CXCL11, and SORT1. Integration of these plasma proteomic signatures with clonal hematopoiesis data improved prediction accuracy, as summarized in Figure 3 (Clinical Benchmarks). These findings are supported by High-strength evidence from the largest included cohort (24), *N* = 427,722, QUIPS Low), with robust multivariable adjustment and Mendelian randomization.

### 3.7 Categorization of evidence by directness

To facilitate interpretation, we categorized the 15 included studies according to the directness of their evidence regarding CHIP biology (Table 4). Only two studies derived evidence directly from defined CHIP cohorts without intervening malignancy (6, 7). Three studies provided CHIP-proximal evidence from early MDS, PV, or MPN cohorts where CHIP represented a precursor state (5, 20, 26). A large longitudinal cohort study tracked the proteomic profiles of individuals with clonal hematopoiesis across varying (VAFs) and risk categories over a 12-year span, alongside individuals at risk for myeloid neoplasms such as (AML), (MDS), and (MPN) (24). The remaining nine studies, while providing valuable mechanistic insights, derived evidence primarily from overt AML, PV, or advanced MDS; these findings require cautious extrapolation to the CHIP-to-malignancy transition and are explicitly identified as such in the synthesis below (4, 8, 9, 18, 19, 21–23, 25).

**Table 4:** Categorization of evidence by directness to CHIP.

Evidence category	Definition	Included studies
Direct CHIP evidence	Studies with defined CHIP cohorts (VAF ≥ 2%, no malignancy)	Ismail et al. (7), Jakobsen et al. (6)
CHIP-proximal evidence	Studies of early MDS/MPN with CHIP as precursor	Zaro et al. (26), Prummel et al. (5), Leimkühler et al. (20)
Extrapolated from overt malignancy	AML/MDS studies providing mechanistic insights into transformation	Raffel et al. (4), Kramer et al. (18), Boer et al. (23), Passaro et al. (9), Moura et al. (19), Rao et al. (21), Amon et al. (22), Weickert et al. (8), Tan et al. (25)
Prognostic/translational	Large cohort studies linking proteomics to disease risk	Tran et al. (24)

### 3.8 Evidence mapping by biological domain

To provide a structured synthesis despite heterogeneity, we mapped findings to four biological domains with corresponding evidence strength:

*Domain 1*: Proteomic Landscape of Aging and Clonal Initiation (Stable/Low-risk CHIP).

Consistent findings: Low mRNA-protein correlation in HSCs (22, 26) Strength: Moderate.

Inconsistent findings: Lineage priming patterns varied between *TET2* and *DNMT3A* mutations—Strength: Moderate.

*Domain 2*: Niche Remodeling (High-risk CHIP, VAF ≥ 5%).

Consistent findings: iMSC emergence with IL-1R1/CD44 expression (5, 8)—Strength: Moderate.

Consistent findings: Loss of hematopoietic support factors (CXCL12, M-CSF, GM-CSF)—Strength: Moderate.

*Domain 3*: Malignant Transformation Drivers (Progression to MDS/AML).

Emerging finding: IL1RAP axis in L-GMP transformation (23)—Strength: low.

Emerging finding: LGALS9-SOCS2 progression markers (25) Strength: low.

*Domain 4*: Plasma Predictive Signatures (Pre-diagnostic/High-risk CHIP).

Consistent finding: F7/IL17RA as causal markers (24) Strength: High.

Emerging finding: S100A8/A9 alarmin complex (20) Strength: Moderate.

## 4 Discussion

### 4.1 Summary of results

This systematic review highlights that the transition from stable CHIP to malignancy is defined by the shift in proteome activity which is invisible to DNA sequencing. Our analysis of the 15 included studies provides strong evidence that plasma proteomics can enhance clinical diagnosis beyond the capabilities of traditional assessments.

A landmark finding by Tran et al. identified 115 proteins associated with incident myeloid neoplasms, specifically validating F7 (Factor VII) and IL17RA as causal markers. This is complemented by Leimkühler et al., who identified the S100A8/S100A9 alarmin complex as a critical systemic biomarker that effectively demarcates the transition from health to disease (20, 24). These plasma predictive findings are supported by high-strength evidence.

A recurring theme across the included studies is the niche remodeling. Weickert et al. demonstrated that bone marrow stromal cells (BMSCs) in MDS and AML patients exhibit a shift toward a “fatty marrow” phenotype, driven by increased adipogenic potential and reduced DLK1 expression (8). Prummel et al. further characterized this by the emergence of inflammatory mesenchymal stromal cells (iMSCs) that expand significantly in MDS, characterized by high expression of IL-1R1 and CD44, which actively suppress healthy hematopoietic support (5). These niche remodeling findings are supported by moderate-strength evidence.

Several studies highlight that mRNA levels often fail to reflect the actual protein landscape, particularly in aging and leukemic stem cells (LSCs). Zaro et al. found that protein diversity increases by 35% in aged HSCs, with the lowest correlation between mRNA and protein (*ρ* = 0.300) found in the HSC compartment (26). Certain studies also identified that LSCs rely heavily on oxidative phosphorylation and specific metabolic regulators like MBOAT7 and CRIP2, which are not always detectable via transcriptomics alone (4, 23). These findings are graded as Moderate strength for the proteogenomic discordance phenomenon but low for the specific metabolic vulnerabilities due to small cohort sizes.

These changes suggest that mutant clones do not just exist; they actively remodel their environment. The remodeling of the bone marrow niche is characterized by a fibrotic extracellular matrix and reduced Notch signaling. This remodeled niche is hostile to healthy cells but protective of mutant ones (27). This explains the significant “proteogenomic discordance” found in the literature: the proteome reflects the cell’s actual fitness and survival strategy, whereas DNA only shows its potential.

### 4.2 The clinical utility of proteomic biomarkers by evidence maturity

To guide clinical translation, we classified biomarkers according to their evidence maturity (Table 5). Markers with established prognostic utility in large cohorts (F7, IL17RA) represent the most clinically advanced candidates, having demonstrated predictive value in >400,000 subjects (24). Validation-phase markers (IL1RAP, iMSC markers) require larger prospective cohorts to confirm prognostic utility (23). Discovery-phase markers (MBOAT7, CRIP2) and therapeutic targets (S100A8/A9, PFKFB3) represent hypothesis-generating findings that require mechanistic and clinical validation before clinical application (4, 20, 21).

**Table 5:** Biomarker classification by evidence level.

Biomarker	Evidence level	Supporting studies	Clinical utility	Validation status
Discovery phase markers				
MPO, NKG7, KLF4	Discovery	Jakobsen et al. (6)	Mechanistic insight	Requires validation
GAR1, DKC1, IDH1	Discovery	Zaro et al., Amon et al. (22, 26)	Understanding aging HSC biology	Requires validation
MBOAT7, CRIP2	Discovery	Raffel et al. (4)	Therapeutic target exploration	Requires validation
Validation phase markers				
IL1RAP	Validation	Boer et al. (23)	Risk stratification marker	Replicated in multiple cohorts
IL-1R1, CD44 (iMSC)	Validation	Prummel et al. (5)	Niche remodeling indicator	Multi-omic validation
S100A8/S100A9	Validation	Leimkühler et al. (20)	Systemic inflammation marker	Validated in MPN cohorts
Prognostic utility markers				
F7, IL17RA	Prognostic	Tran et al. (24)	Incident MN risk prediction	Validated in 427,722 subjects
BIN2, FGF2, LY9, CXCL11, SORT1	Prognostic	Tran et al. (24)	Risk score components	Requires prospective validation
LGALS9, SOCS2	Prognostic	Tan et al. (25)	Progression monitoring	Requires larger cohort validation
Therapeutic potential markers				
S100A8/S100A9	Therapeutic	Leimkühler et al. (20)	Tasquinimod target	Preclinical validation
IL1RAP	Therapeutic	Boer et al. (23)	Immunotherapy target	Preclinical
MBOAT7, CRIP2	Therapeutic	Raffel et al. (4)	Metabolic vulnerability	Preclinical
PFKFB3	Therapeutic	Rao et al. (21)	Glycolytic inhibitor target	Preclinical

Identifying specific progression markers moves the clinical conversation from reactive diagnosis to proactive interception. The findings highlight that changes in proteins such as LGALS9 and SOCS2 are not random fluctuations but are functional checkpoints in myeloid evolution. This is clearly shown by Tan et al. which tracked LGALS9 and SOCS2 proteomes throughout the different stages of the disease progression (25). Their findings concluded that LGALS9 and SOCS2 increased as the disease got worse. Rather than waiting for a rise in Variant Allele Frequency (VAF) or the onset of cytopenia, these proteomic changes could serve as early warning signs. Table 6 summarizes the prognostic significance of various proteomic markers and their correlation with the stage of the disease. These findings suggest the potential for a more proteomics-informed approach to myeloid oncology, though prospective validation in clinical settings is required before such a model can be implemented. Integrating high-sensitivity proteomics into the standard monitoring of high-risk CHIP cohorts allows for a stratified approach, identifying patients destined for the malignant transition months or years before traditional clinical markers manifest.

**Table 6:** Disease stage and clinical implications of the proteomics markers.

Stage of progression	Proteome/Protein	Status	Effect on transformation and niche
Stage 1: clonal initiation	MPO, NKG7, KLF4	Upregulated (expressed protein)	Drives expression of mature myeloid proteins, indicating distinct myeloid lineage-associated priming in *TET2* mutant CHIP (6).
	CD38, CD45RA	Upregulated (increased)	Associated with *TET2* mutations and mature myeloid lineage priming (6).
	CD49f	Downregulated (reduced)	Reduced levels are associated with *TET2* mutation priming (6).
Stage 2: niche remodeling (VAF ≥ 5%)	IL-1R1, CD44	Upregulated (increased)	High expression defines the emergence of inflammatory mesenchymal stromal cells (iMSCs), shifting the niche to a dysfunctional state (5).
	CXCL12, M-CSF, GM-CSF	Downregulated (reduced)	Loss of these key support proteins accompanies the shift from a regenerative to a dysfunctional stromal program (5).
	MCP-1	Upregulated (elevated in serum)	Elevated in serum, associated systemically with the *DNMT3A*-mutant CHIP microenvironment (7).
Stage 3: progression from CHIP to malignancy	IL1RAP	Upregulated (increased)	Marks a leukemic GMP-like state; represents the critical boundary between clonal expansion and leukemic transformation (23).
	IL-8, CXCL1	Upregulated (secreted)	Inflammatory mediators secreted due to IL1RAP activation; reduces proliferation/colony-forming capacity of healthy CD34 + cells while favoring leukemic expansion (23).
Stage 4: definitive leukemic evolution (MDS/AML)	LGALS9, SOCS2	Upregulated (increased)	Functional checkpoints activating in HSC/MPP populations; marks the definitive transition toward myeloid evolution. They further increase as the disease gets worse (25).
	DLK1	Downregulated (reduced expression)	Reduced expression drives bone marrow stromal cells (BMSCs) to shift toward a “fatty marrow” phenotype with increased adipogenic potential (8).
	MBOAT7, CRIP2	Upregulated (relied upon)	Specific metabolic regulators heavily relied upon by Leukemic Stem Cells (LSCs) for oxidative phosphorylation (4).
Systemic predictive markers (pre-diagnosis)	F7, IL17RA	Upregulated (detected)	Causal plasma protein markers associated with incident myeloid neoplasms and disease progression more than a decade prior (24).
	S100A8/S100A9	Upregulated (detected)	Alarmin complex that acts as a critical systemic biomarker effectively demarcating the transition from health to disease (20).
	BIN2, FGF2, LY9, CXCL11, SORT1	Upregulated (detected)	Plasma proteins that are associated with future myeloid neoplasm risk (24).

### 4.3 Functional mechanisms of key proteomic drivers

Beyond their identification as biomarkers, several proteomic candidates possess established or emerging functional roles in clonal expansion. IL1RAP, which marks the leukemic granulocyte-monocyte progenitor state, potentiates IL-1 signaling and activates p38 MAPK and NF-κB pathways within the inflammatory niche (23). LGALS9 (galectin-9) suppresses anti-tumor immunity via TIM-3 engagement and promotes myeloid progenitor self-renewal through co-activation of NF-κB and *β*-catenin stabilization (28). SOCS2, a negative regulator of cytokine signaling, can modulate therapeutic responses, as its loss enhances interferon-mediated depletion of JAK2-mutant stem cells (29). The S100A8/A9 alarmin complex directly activates TLR4 on mesenchymal stromal cells, driving myofibroblast differentiation and migration (30). Systemically, F7 (coagulation factor VII) promotes inflammatory myeloid cell recruitment through PAR-2-mediated signaling, while IL17RA links adaptive immune dysregulation to chronic niche injury (31). Collectively, these proteins are not passive markers but active participants in clonal evolution. However, the causal role of many of these proteins in CHIP progression remains to be definitively established through functional studies in CHIP-specific models.

### 4.4 Genotype-specific proteomic programs: divergent pathways and knowledge gaps

Our synthesis reveals distinct proteomic consequences of different CHIP-driving mutations as discussed below:

*DNMT3A* mutations: Associated with elevated IL-32 in lymphoid cells, increased serum MCP-1, and epigenetic priming affecting both myeloid and lymphoid lineages (7). *DNMT3A*-mutant CHIP is also linked to niche remodeling at VAF ≥ 5% (5).

*TET2* mutations: Drive expression of mature myeloid proteins (MPO, NKG7, KLF4), altered surface marker expression (↑CD38/CD45RA, ↓CD49f), and attenuated inflammatory responses that may confer a selective advantage (6).

*JAK2V617F*: Induces systemic metabolic alterations in MPN context (21), drives S100A8/A9-mediated myelofibrosis (20).

*ASXL1, SRSF2,* and *TP53* mutations: Critically, no included study provided proteomic profiling specifically of *ASXL1*-, *SRSF2*-, or *TP53*-mutant CHIP. This represents a major knowledge gap, as these mutations are associated with higher leukemic transformation risk and distinct biological programs (*ASXL1*: chromatin dysregulation; *SRSF2*: RNA splicing; *TP53*: genomic instability) (32–35). The proteomic consequences of these high-risk genotypes cannot be inferred from *DNMT3A/TET2* data (35, 36). This “genotype-proteotype gap” is identified as a priority for future research, requiring recruitment of genotypically stratified CHIP cohorts with dedicated deep proteomic profiling.

### 4.5 The missing spatial architecture: emerging platforms

All 15 included studies relied on bulk or single-cell suspension proteomics, losing the spatial context of cell–cell interactions. Recent advances in spatial proteomics—including Imaging Mass Cytometry (IMC), CODEX, MIBI-TOF, and laser capture microdissection coupled to ultra-low-input mass spectrometry—have begun to map the human bone marrow niche at subcellular resolution. In AML and myeloproliferative neoplasms, spatial proteomics has revealed that leukemic stem cells localize to distinct peri-sinusoidal niches with endothelial and mesenchymal signatures (27, 37). In healthy aging, spatial mapping shows progressive loss of CXCL12 + reticular cells and emergence of inflammatory fibroblasts (37). Applying these platforms to CHIP bone marrow biopsies would directly visualize the physical interactions between mutant clones and iMSCs, identifying whether clonal expansion is spatially restricted or diffuse. Based on our synthesis, spatial proteomic mapping represents a priority area for future investigation to address the current gap in understanding the physical organization of clonal expansion (38).

### 4.6 Limitations and methodological considerations

Several limitations warrant consideration. First, the absence of prospective protocol registration (e.g., PROSPERO) introduces potential for reporting bias, despite our adherence to a pre-specified internal protocol and PRISMA 2020 guidelines. This limitation is inherent to our review design and should be considered when interpreting our findings. Secondly, the exclusion of Embase and Web of Science due to institutional access limitations may have resulted in the omission of relevant studies; however, we supplemented our search with Google Scholar and manual reference list curation to mitigate this risk. Third, the substantial methodological heterogeneity across studies precluded meta-analysis and necessitated a narrative synthesis approach.

### 4.7 Cautions regarding novel terminology and models

While we introduce the concept of a “functional niche failure” to integrate our findings, this term represents interpretative models derived from the available evidence rather than established biological paradigms. Future mechanistic studies are needed to validate whether the proteomic changes we describe represent causal drivers.

### 4.8 Grading of evidence strength

To facilitate interpretation of our findings, we graded the strength of evidence for key conclusions based on study design, sample size, risk of bias, and consistency across studies using the GRADE framework (39, 40). Detailed grading of evidence strength is presented in Table 7. Overall strength is categorized as High, Moderate, Low, or Very Low, with downgrades applied primarily for imprecision (limited sample sizes) or moderate risk of bias (40).

**Table 7:** Grade of evidence strength.

Finding	Strength of evidence	Supporting studies	Rationale
*DNMT3A*-mutant CHIP associated with IL-32/MCP-1 dysregulation	Moderate	Ismail et al. (*N* = 243, ROBINS-E Low) (7)	Single well-powered cohort with mechanistic validation
iMSC emergence in CHIP/MDS	Moderate	Prummel et al. (*N* = 84, ROBINS-E Low) (5)	Limited sample size but multi-omic validation
LSC metabolic dependencies (MBOAT7, CRIP2)	low	Raffel et al. (*N* = 26, ROBINS-E Moderate) (4)	Small sample, requires replication
F7/IL17RA as causal plasma markers	High	Tran et al. (*N* = 427,722, QUIPS Low) (24)	Large biobank with MR and validation
LGALS9-SOCS2 progression axis	low	Tan et al. (*N* = 39, QUIPS Moderate) (25)	Small cohort but functional validation
Proteogenomic discordance in aging HSCs	moderate	Zaro et al. (mouse), Amon et al. (*N* = 11) (26)	Consistent across species, though human sample small

*DNMT3A*-mutant CHIP and IL-32/MCP-1 dysregulation was graded as Moderate strength. Although Ismail et al. utilized a well-powered observational design ( N=243 ) with low risk of bias using the ROBINS-E tool (7), the rating was restricted to moderate due to the reliance on a single primary cohort lacking broader cross-validation. iMSC emergence in CHIP/MDS was rated as Moderate strength based on Prummel et al. ( N=84 , ROBINS-E Low) (5). Despite a limited sample size that introduces potential imprecision, the strength was sustained at a moderate level due to robust multi-omic methodological validation. LSC metabolic dependencies (MBOAT7, CRIP2) received a Low strength score. Data from Raffel et al. ( N=26 , ROBINS-E Moderate) featured a small sample size and moderate risk of bias, resulting in serious imprecision that necessitates independent replication (4). F7/IL17RA as causal plasma markers achieved a High strength rating. Tran et al. evaluated a massive biobank cohort ( N=427,722 , QUIPS Low) (24), combining rigorous prognostic quality appraisal with Mendelian Randomization and independent validation, minimizing risk of bias and imprecision. LGALS9-SOCS2 progression axis, which was evaluated specifically as an indicator of active malignant progression to leukemia differentiating it from stable clonal hematopoiesis, was assessed as Low strength. While Tan et al. ( N=39 , QUIPS Moderate) provided valuable functional validation for malignant progression, the small cohort size and moderate risk of bias led to downgrades (25). Proteogenomic discordance in aging HSCs was graded as Moderate strength. Although Amon et al. evaluated a very small human sample ( N=11 ) (22), direct biological consistency with Zaro et al.’s murine model data mitigated further downgrading for imprecision, balancing cross-species validity (26). Strong conclusions were reserved for findings supported by large, well-controlled cohorts (e.g., Tran et al.) or consistent multi-species evidence (e.g., proteogenomic discordance) (24). Findings derived from small mechanistic cohorts (e.g., Raffel et al., Kramer et al.) are presented as hypothesis-generating observations requiring validation (4, 18).

### 4.9 Limitations of small sample sizes in deep proteomic studies

A notable tension in the included literature exists between technical sophistication and statistical power. While platforms such as DIA-MS, TMT-labeling, and Olink panels provide unprecedented proteomic depth, many studies employed limited sample sizes (e.g., Raffel et al. *n* = 26, Amon et al. *n* = 11, Tan et al. *n* = 39) (4, 22, 25). These small cohorts, while justified by the intensive resource requirements of deep proteomics and the rarity of well-characterized CHIP specimens, constrain the generalizability and reproducibility of findings. Specifically, small sample sizes: (1) increase the risk that identified signatures reflect individual biological variance rather than disease-specific signals; (2) limit the ability to adjust for key confounders (age, sex, comorbidities); and (3) restrict subgroup analyses by mutation type. For example, the moderate ROBINS-E ratings assigned to five studies primarily reflected concerns about confounding due to restricted sample sizes (4, 8, 18, 19, 23).

Conversely, studies employing proteomic platforms amenable to high-throughput screening (e.g., Olink panels) demonstrated that biomarker discovery can be conducted at scale when using targeted or proximity-extension assays rather than discovery mass spectrometry. Tran et al. analysis of 427,722 UK Biobank participants exemplifies how plasma proteomics can achieve statistical robustness while sacrificing some proteomic depth (24). Future research should prioritize: (1) multi-center validation of discovery-phase proteomic signatures in larger cohorts; (2) development of targeted assays (PRM, MRM) to enable validation of mass spectrometry findings at scale; and (3) establishment of biobanks with well-phenotyped CHIP specimens to facilitate adequately powered proteomic studies.

## 5 Conclusion

This systematic review demonstrates that the fitness of a mutant clone is inextricably linked to its ability to remodel the bone marrow microenvironment. By synthesizing evidence across multiple biological compartments, we reveal that genomic data alone is insufficient to capture the inflammatory and metabolic shifts driving transformation. However, the field has reached a critical bottleneck: spatial context is missing, high-risk genotypes (*ASXL1, SRSF2*) are largely unstudied, and longitudinal proteomic kinetics during active transition are absent. Future research must prioritize spatial proteomic platforms (IMC, CODEX, and spatial MS) and genotype-stratified longitudinal cohorts to move beyond genomic surveillance toward functional, protein-based risk stratification and early clinical interception.

## Data Availability

The original contributions presented in the study are included in the article/supplementary material, further inquiries can be directed to the corresponding author.

## Glossary

CHIP: Clonal hematopoiesis of indeterminate potential
DIA-MS: Data-independent acquisition mass spectrometry
TMT: Tandem Mass tags
HSPCs: Hematopoietic stem/progenitor cells
mRNA: Messenger ribonucleic acid
iMSCs: Inflammatory mesenchymal Stromal cells
BM: Bone marrow
MDS: Myelodysplastic syndrome
AML: Acute myeloid leukemia
GoTChA: Genotyping of Targeted loci with single-cell Chromatin Accessibility
NGS: Next-Generation Sequencing
NK cells: Nature killer cells
VAF: Variant Allele Fraction
CFU-F: colony-forming unit-fibroblast
IL1RAP: interleukin-1 receptor accessory protein
L-GMP: leukemic granulocyte–monocyte progenitor
BMSCs: bone marrow stromal cells
LSCs: leukemic stem cells
PV: Polycythemia Vera
RoB: Risk of bias
QUIPS: Quality in prognosis studies
ROINS-E: Risk of bias in Nonrandomized studies-of exposure
SYRCLE: Systematic Review Centre for Laboratory animal Experimentation
MeSH: Medical subject headings
LFQ: Label-Free Quantification
TARGET: TARGETed high-sensitivity single-cell mutational analysis with parallel RNA SEQuencing
HSC: Hematopoietic stem cell
MN: Myeloid neoplasm
GM-CSF: granulocyte–macrophage colony-stimulating factor
M-CSF: macrophage colony-stimulating factor
TEMRA cells: Terminally differentiated Effector Memory cells re-expressing CD45RA
CFU-F: Colony-forming unit-fibroblast
NES-high: Nestin high
AUC: Area under curve
DNA: Deoxyribonucleic acid
MPP: Multipotent Progenitor Proteomics
CXCL12: Chemokine C-X-C motif ligand 12
GM-CSF: granulocyte–macrophage colony-stimulating factor
BIN2: Bridging Integrator 2
FGF2: Fibroblast growth factor 2
LY9: Lymphocyte Antigen 9
CXCL11: Chemokine C-X-C motif ligand 11
SORT1: Sortilin
IL1RAP: Interleukin 1 receptor accessory protein
GAR1: Glycine and arginine rich protein 1
DKC1: Dyskerin pseudouridine synthase 1
IDH1: Isocitrate dehydrogenase 1
CRIP2: Cysteine-rich intestinal protein 2
CXCL1: Chemokine C-X-C motif ligand 1
F7: Factor 7
S100A8/S100A9: S100 calcium-binding protein A8/S100 calcium-binding protein A9
SOCS2: Suppressor of cytokine signaling 2
NKG7: Natural killer cell granule protein 7
MPO: Myeloperoxidase
M-CSF: macrophage colony-stimulating factor
MBOAT7: Membrane bound O-acyltransferase domain containing 7
MCP-1: Monocyte chemoattractant protein-1
LGALS9: Lectin galactose binding soluble 9
KLF4: Krüppel like factor 4
IL-8: Interleukin 8
IL-32: Interleukin 32
IL-1R1: Interleukin 1 receptor 1
IL-1RAP: Interleukin 1 receptor accessory protein
IL17RA: Interleukin 17 receptor A
DLK1: Delta-like non-canonical Notch ligand 1
iMSC: inflammatory mesenchymal stromal cell
L-GMP: leukemic granulocyte-monocyte progenitor
